# Robert Remak (1815–1865)

**DOI:** 10.1007/s00415-012-6761-6

**Published:** 2012-11-28

**Authors:** Andrzej Grzybowski, Krzysztof Pietrzak

**Affiliations:** 1 Department of Ophthalmology, Poznan City Hospital, ul. Szwajcarska 3, 61-285 Poznań, Poland; 2 Department of Ophthalmology, University of Warmia and Mazury, Olsztyn, Poland; 3 Department of Orthopaedics and Traumatology, University of Medical Sciences, Poznań, Poland

 Robert Remak was a neurologist, a physiologist, and an embryologist. He was born on July 23, 1815 in Poznań; this town and a large western part of Poland was occupied by Prussia during his lifetime. In his papers, written in Polish, he refers to Poles as his compatriots [3]. Later, when Prussian rule dictated that his further career depended on receiving baptism, he refused to reject his Jewish identity.

Remak started his education at home, subsequently completed a Polish gymnasium in Poznań and in 1833 enrolled at the University of Berlin. While still an undergraduate, he started research work in the microscopic laboratory under Johannes Müller (1801–1858), professor of anatomy and physiology. The fruit of his many years’ work was a doctoral dissertation entitled “Observationes Anatomicae et Microscopicae de Systematis Nervosi Struktura” [2], which he defended in 1838. In his thesis, he not only described axial fibers encased in myelin sheaths, but he was the first to describe unmyelinated (sympathetic) nerve fibers, called Remak’s fibers. He demonstrated that the grey color of these fibers results from a lack of a myelin sheath. He also discovered that fibers from motor neurons in the spinal cord course without interruption in the anterior roots and peripheral nerves. Later, Purkyne named these connections “axis cylinders”. Remak further showed in his thesis that sympathetic ganglia are of great importance in the functioning of the nervous system [2].

After the doctorate, he continued neurological investigations in Müller’s lab. In 1839, he described ganglion cells in the right atrium and related them to the sympathetic system [4]. To date, this is called Remak’s ganglion [1]. In 1844, he was the first to demonstrate that the cerebral cortex consists of six layers [7]. Between 1843 and 1844 he established the presence of extremely fine fibrils within the axis cylinder, contrary to the common belief that the inside of nerves was either empty or filled with fluid [6].

In 1843, he became assistant in the clinic of Johann Schönlein (1793–1864) at the Charité Hospital. He rejected the offer of a chair in Vilnius and Cracow, by referring to the scope of his scientific work in Berlin. However, according to legal provisions at that time, as a Jew he was banned from occupying the post of professor in areas under Prussian rule. It was only in 1847 that he obtained an (unpaid) position of extramural lecturer (Privatdozent) at Berlin University, after many efforts and letters of support of Schönlein and Alexander von Humboldt (1769–1859). He still continued clinical work at the Charité. In 1859, he was promoted to the post of assistant professor, which was a disproportionately humble position in view of his scientific achievements.

Apart from his neurological discoveries, embryology was an important field of interest. His research in this area continued over 20 years in a small laboratory at Charité Hospital. He was the first to assert that there are three germ layers in the early embryo, and that new cells are generated by division of the existing ones [8]. Additionally, he delved into the fields of dermatology and cancerous changes [5]. At the Charité, he carried out research on animals by cutting their nerves; he then stimulated the remaining nerve endings with electrical current and observed the return of the damaged function, thus determining the scope of activity particular nerves [9]. According to Remak, this method was a double proof of the function of nerves.

From 1856 onwards, Remak had a private practice, largely based on electrotherapy, another field of interest. His patients were treated with galvanotherapy. The method was applied in about 700 patients, especially those affected with diseases of the brain and spinal cord. Remak determined the point where the nerves penetrated the muscles, which was essential for effective electrical stimulation. In contrast to other physicians, he therefore did not stimulate muscle tissue, but nerve terminals in muscle [10]. His method became the subject of priority disputes, especially with Duchenne de Boulogne (1806–1875). He also constructed an electrotherapy apparatus that was commonly used and dubbed “Remak’s apparatus”.

In 1847, Remak married Feodowa Mayer. They had a son, Ernest Juliusz Remak (1849–1912), who also became a neurologist [1]. Robert Remak’s grandson, Robert Remak (1888–1942), was a mathematician, and was murdered by the Nazis in Auschwitz. The great neuroscientist Robert Remak died on August 29, 1865, in Bad Kissingen, Germany.
